# Association of Serum Testosterone Levels With Angiographic Severity and Six-Month Outcomes in Coronary Artery Disease in Young and Middle-Aged Men: A Prospective Observational Study From North India

**DOI:** 10.7759/cureus.97663

**Published:** 2025-11-24

**Authors:** Ashish Jha, Sandeepan Saha, Ejaz Shams, Bhuwan C Tiwari, Sudarshan K Vijay, Naveen Jamwal, Amresh Kumar Singh, Arvind Kumar Singh, Manish Kulshrestha

**Affiliations:** 1 Cardiology, Dr. Ram Manohar Lohia Institute of Medical Sciences, Lucknow, IND; 2 Community Medicine, Dr. Ram Manohar Lohia Institute of Medical Sciences, Lucknow, IND; 3 Biochemistry, Dr. Ram Manohar Lohia Institute of Medical Sciences, Lucknow, IND

**Keywords:** coronary angiographic severity, coronary artery disease, mace, syntax score, testosterone

## Abstract

Background

Coronary artery disease (CAD) remains a major health challenge worldwide, with a rising burden in India. While traditional risk factors are well recognized, the influence of testosterone deficiency on CAD severity is not clearly established in the Indian population.

Objectives

The objective of this study is to evaluate the association between serum testosterone levels and the angiographic severity of CAD, assessed by the SYNTAX score (SS), in men aged 20-60 years with CAD. The secondary objectives were to assess the impact of testosterone on major adverse cardiovascular events (MACE) at six months and to examine correlations with clinical profile and CAD risk factors.

Methods

This prospective observational study included 269 male patients undergoing coronary angiography at a tertiary care center. Serum total and free testosterone were measured before angiography. The patients were stratified by SYNTAX score (<23 versus ≥23), clinical presentation (stable CAD versus acute coronary syndrome {ACS}), and age (20-40 versus 41-60 years). Follow-up for MACE was performed at six months.

Results

Serum testosterone levels did not differ significantly between patients with mild and moderate/severe CAD (total testosterone: 327.49 ± 160.48 ng/dL versus 336.03 ± 167.75 ng/dL; free testosterone: 28.40 ± 14.24 pg/mL versus 29.35 ± 15.48 pg/mL). Levels were comparable across age and clinical subgroups. Testosterone values were lower among diabetic patients but showed no consistent association with other risk factors. At six months, MACE occurred in 3.3% of the patients, without a significant relationship to testosterone levels.

Conclusion

Serum testosterone levels were not significantly related to angiographic severity or short-term outcomes in this cohort. Routine testosterone testing may have limited value for CAD risk assessment in this population.

## Introduction

Coronary artery disease (CAD) remains the leading cause of mortality and morbidity worldwide, accounting for over nine million deaths annually [1]. India faces a disproportionately high and rising burden of CAD, driven by rapid urbanization, lifestyle transitions, and the increasing prevalence of traditional risk factors such as hypertension, diabetes mellitus, dyslipidemia, obesity, and smoking. In addition to these biological and behavioral contributors, social determinants of health, such as socioeconomic status, education, and access to healthcare, are increasingly recognized as key influences on cardiovascular risk.

Beyond these established risk factors, there is a growing recognition of nontraditional contributors to atherosclerosis, particularly endocrine factors such as testosterone deficiency. Low serum testosterone has been associated with metabolic syndrome, insulin resistance, visceral adiposity, and a pro-inflammatory milieu, all of which may accelerate atherogenesis [2]. Several studies have reported an inverse relationship between testosterone levels and CAD severity. Phillips et al. first demonstrated that men with angiographically confirmed CAD had lower testosterone levels, independent of age and adiposity [3]. Subsequent Indian studies by Gururani et al. [4] and Gopalakrishnan et al. [5] also found lower testosterone levels in patients with CAD compared with controls and highlighted a potential endothelial protective role of testosterone.

Given the limited and heterogeneous data from Indian populations, we sought to investigate whether serum testosterone levels are associated with the angiographic severity of CAD. The primary objective was to determine the relationship between serum testosterone and CAD severity, as assessed by the SYNTAX score (SS), in young and middle-aged Indian men (20-60 years). Secondary objectives included evaluating the association of testosterone with major adverse cardiovascular events (MACE), a composite of cardiovascular death, non-fatal myocardial infarction (MI), target vessel revascularization, and stroke, at six months and exploring its correlation with clinical profiles and conventional risk factors.

## Materials and methods

Study design and participants

This was a prospective, single-center observational study conducted in the Department of Cardiology at a tertiary care teaching institute in North India over two years. Consecutive male patients aged 20-60 years who were diagnosed with coronary artery disease (stable CAD or acute coronary syndrome {ACS}) and were scheduled for clinically indicated coronary angiography were invited to participate. Consecutive sampling was used to minimize selection bias. Patients meeting the inclusion criteria and providing written informed consent were enrolled. Recruitment was done from both inpatient and outpatient cardiology services to ensure a representative clinical spectrum. Exclusion criteria included age outside 20-60 years, previous revascularization, normal coronary arteries, myocarditis, cardiomyopathy, pulmonary embolism, the use of testosterone supplementation or anti-androgen therapy, prostate cancer, significant hepatic or renal dysfunction, current or recent infection, or refusal to consent.

Sample size calculation

The study was designed to test the hypothesis that the proportion of patients with moderate/severe CAD is higher among those with low serum testosterone compared to those with normal testosterone. A chi-square test was planned to assess the association between testosterone category (low versus normal) and CAD severity (mild versus moderate/severe). Using estimates from Li et al., the prevalence of moderate/severe CAD was assumed to be 41.1% among patients with normal testosterone and approximately 58.7% among those with low testosterone, corresponding to an odds ratio of about 2.0 [6].

Based on these assumptions, with a 95% confidence level and a precision of ±10% around the estimated proportions, the required minimum sample size was calculated to be 66 participants. Allowing for an anticipated 10% loss of data, the target was increased to 74 participants.

Because the study aimed to compare two clinical presentations (stable CAD and ACS) across two age groups (20-40 years and 41-60 years), the required sample was multiplied by four, yielding a total target of 296 patients. Due to challenges in recruiting stable patients with CAD in the younger age group, 269 participants were ultimately enrolled. The study was approved by the Institutional Ethics Committee of Dr. Ram Manohar Lohia Institute of Medical Sciences (approval number: 52/22).

Baseline assessment and data collection

After written informed consent, clinical history, cardiovascular risk factors, and symptoms of decreased libido or erectile dysfunction were documented. Physical examination and baseline tests (complete blood count {CBC}, renal/liver function, lipid profile, blood glucose, and ECG) were performed, and body mass index (BMI) was calculated. Standard criteria were used to define hypertension, diabetes, dyslipidemia, smoking, and family history of premature CAD.

Testosterone measurement and coronary angiography

Two milliliters of fasting venous blood were collected between 8:00 and 10:00 a.m. in a plain vacutainer for serum testosterone estimation. Samples were centrifuged at 3000 revolutions per minute (rpm) for 10 minutes, and serum was analyzed immediately. Serum total and free testosterone were measured using an electrochemiluminescence immunoassay (ECLIA) on the Cobas 6000 analyzer (Roche Diagnostics, Mannheim, Germany), employing the manufacturer’s commercial kits. The assay had been previously validated in-house according to laboratory quality control procedures, with both intra-assay and inter-assay coefficients of variation below 10%. Calibration and two-level internal quality controls were run daily as per manufacturer recommendations.

The reference ranges used for adult men in our laboratory were as follows: total testosterone: 300-1000 ng/dL and free testosterone: 9-30 pg/mL. Values below these thresholds were considered low for the purpose of subgroup analysis.

Coronary angiography was done via radial or femoral access, and the SYNTAX score (SS) was calculated for each patient. Patients with SS of <23 were classified as mild (mild CAD) and those with SS of ≥23 as moderate/severe (moderate/severe CAD).

Blinding and reliability

SYNTAX scoring was performed independently by two experienced interventional cardiologists who were blinded to the patients’ testosterone levels and clinical details. In cases of discrepancy exceeding five points between the two observers, a consensus score was assigned after joint review. The inter-observer reliability for SYNTAX scoring in a subset of 50 randomly selected cases showed a high level of agreement (intraclass correlation coefficient {ICC} = 0.91), indicating excellent consistency.

Follow-up and statistical analysis

The patients were followed for six months to record major adverse cardiovascular events (MACE), defined as a composite of cardiovascular death, non-fatal myocardial infarction, target vessel revascularization, and stroke, via clinic visits or phone calls.

Continuous variables were expressed as mean ± standard deviation (SD) or median (interquartile range), depending on data distribution, while categorical variables were presented as frequencies and percentages. The normality of continuous variables was assessed using the Shapiro-Wilk test. For normally distributed variables, comparisons between two groups were performed using an independent samples t-test; for non-normally distributed variables, the Mann-Whitney U test was used. Categorical variables were analyzed using the chi-square test or Fisher’s exact test, as appropriate.

Correlations between continuous variables (e.g., testosterone levels and SYNTAX score) were evaluated using Pearson’s correlation coefficient for normally distributed data and Spearman’s rank correlation coefficient for non-normal data. A two-sided p-value of <0.05 was considered statistically significant.

Missing data were addressed using complete-case analysis. Patients with missing key variables, including testosterone levels or SYNTAX score, were excluded from the respective analyses, and the proportion of missing data was reported. Sensitivity analyses were performed where appropriate to ensure the robustness of results. All statistical analyses were performed using SPSS version 21.0 (IBM Corp., Armonk, NY).

## Results

Study population and baseline characteristics

A total of 269 male patients with CAD aged 20-60 years were included in the final analysis. The participants were stratified based on angiographic severity, age (20-40 and 41-60), and clinical presentation, stable CAD or ACS. SYNTAX score was used to classify patients into two groups: 124 patients (46.1%) with scores of <23 (mild CAD) and 145 patients (53.9%) with scores of ≥23 (moderate/severe CAD). Baseline clinical and demographic characteristics of the study population are summarized in Table 1, which were largely comparable between the two groups. Patients with moderate/severe CAD showed a non-significant trend toward higher age (p = 0.08) and lower left ventricular ejection fraction (p = 0.07).

**Table 1:** Baseline clinical and demographic data BMI, body mass index; SBP, systolic blood pressure; DBP, diastolic blood pressure; HR, heart rate; LVEF, left ventricular ejection fraction; SD, standard deviation; CAD, coronary artery disease

Variable	Overall (mean ± SD)	Mild CAD (mean ± SD)	Moderate/severe CAD (mean ± SD)	P-value	T-value
		(N = 124; 46.1%)	(N = 145; 53.9%)		
Age	48.21 ± 8.57	47.23 ± 8.50	49.06 ± 8.57	0.08	-1.75
BMI	26.06 ± 4.00	26.18 ± 4.19	25.94 ± 3.85	0.62	0.49
SBP (mm Hg)	129.5 ± 15.3	128.3 ± 16.30	130.5 ± 14.30	0.23	-1.17
DBP (mm Hg)	76.1 ± 10.71	76.3 ± 9.80	76 ± 11.40	0.81	0.23
HR (beats/minute)	76.58 ± 10.48	77.44 ± 10.50	75.83 ± 10.46	0.21	1.26
LVEF (%)	49.63 ± 10.18	50.81 ± 9.63	48.63 ± 10.55	0.07	1.77
Syntax score	23.17 ± 11.41	13.75 ± 5.70	31.22 ± 8.54		

Clinical presentation and risk factors

Acute coronary syndrome was the predominant presentation, with anterior wall ST elevation myocardial infarction (STEMI) being most common, followed by Inferior wall myocardial infarction (IWMI) and non-STEMI (NSTEMI); unstable angina was rare. The distribution of ACS versus stable CAD was similar across SYNTAX score groups (p = 0.26; Table 2). Traditional cardiovascular risk factors, including diabetes, hypertension, smoking, dyslipidemia, family history of CAD, and diet preference, were also similar between groups (p > 0.05). The duration of symptoms was modestly longer in the higher SYNTAX score group (p = 0.038).

**Table 2:** Presenting diagnosis, symptom duration, and risk factors CAD, coronary artery disease; ACS, acute coronary syndrome; AW, anterior wall; IW, inferior wall; LW, lateral wall; STEMI, ST elevation myocardial infarction; USA, unstable angina; DM, diabetes mellitus; HTN, hypertension; F/H, family history; Dx, diagnosis

Variable	Value (proportion {%})	Mild CAD (%)	Moderate to severe CAD (%)	P-value	Calculated χ²
Dx: stable CAD	101 (37.55)	50	51	0.26	1.26
ACS	168 (62.45)	74	94	0.26	0.18
USA	1 (0.37)	0	1		
NSTEMI	37 (13.75)	19	18		
AW STEMI	62 (23.05)	31	31		
IW STEMI	56 (20.82)	21	35		
LW STEMI	12 (4.46)	3	9		
Symptom duration					
<1 month	96 (35.69)	54	42		1.79
One month to one year	106 (39.40)	41	65	0.03	5.47
>1 year	67 (24.90)	29	38		0.88
Risk factors: smoking	105 (39.03)	51	54	0.53	0.40
DM	80 (29.74)	39	41	0.38	0.76
HTN	65 (24.16)	26	39	0.31	1.03
Dyslipidemia	24 (8.92)	8	16	0.21	1.59
F/H CAD	74 (27.51)	33	41	0.79	0.07
Diet (non-vegetarian)	162 (60.22)	77	85	0.62	0.24

Testosterone levels and angiographic severity

**Figure 1:**
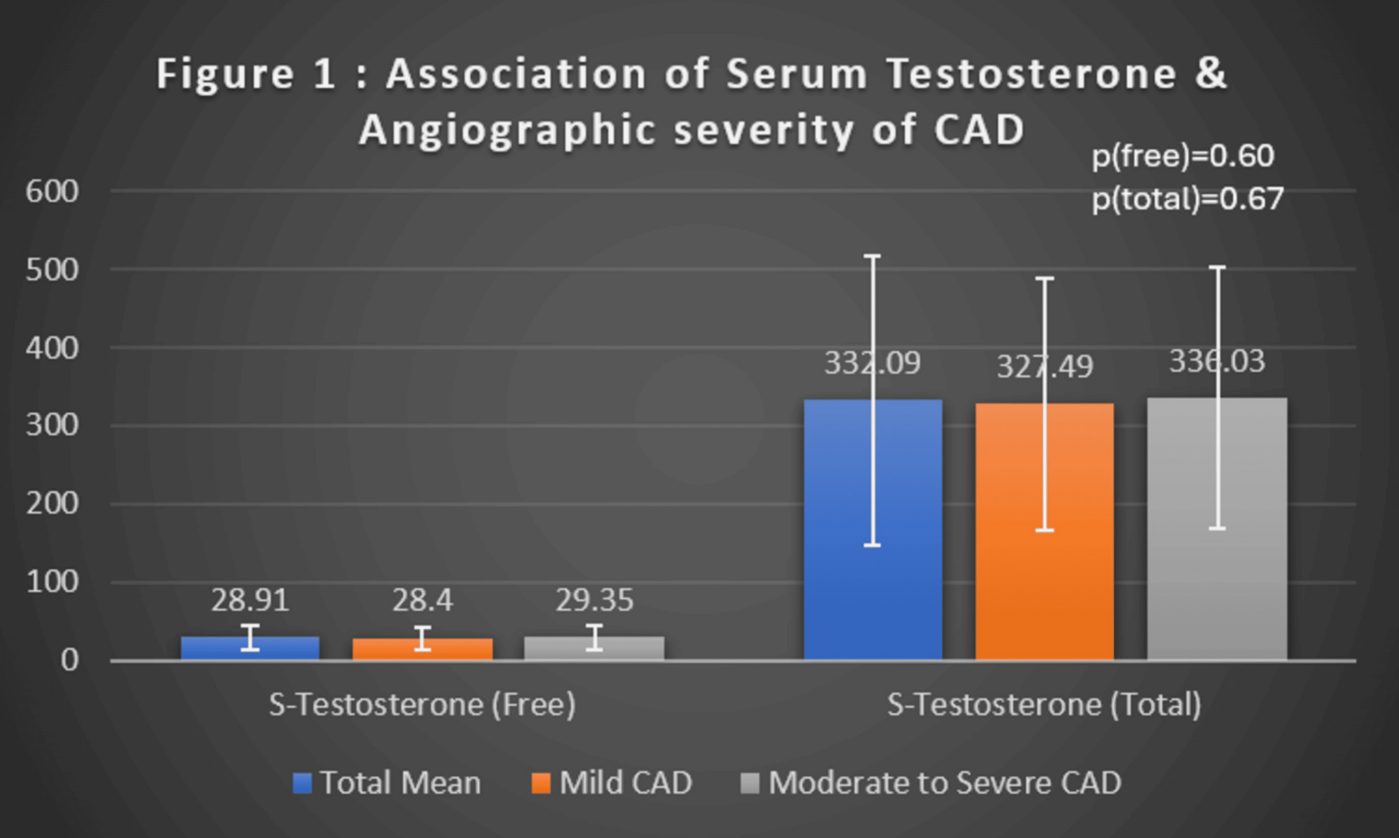
Association of serum testosterone and angiographic severity of CAD CAD: coronary artery disease

As shown in Figure 1, the mean total testosterone was 327.49 ± 160.48 ng/dL in the mild CAD group and 336.03 ± 167.75 ng/dL in the moderate/severe group (p = 0.67), while the mean free testosterone was 28.40 ± 14.24 pg/mL and 29.35 ± 15.48 pg/mL (p = 0.60), respectively, with no significant difference between the groups. Correlation analyses further confirmed the lack of association between testosterone levels and angiographic severity. Pearson’s correlation coefficient between total serum testosterone and SYNTAX score was r = -0.0008, while for free testosterone, it was r = -0.0179, demonstrating negligible and statistically non-significant relationships.

Age-stratified and clinical subgroup analysis

The correlation between age subgroups and the presentation of CAD and angiographic severity is summarized in Table 3. When comparing clinical presentation across age groups, ACS was more prevalent among those aged 41-60 years. However, this difference was not statistically significant. Similarly, the distribution of angiographic severity was comparable between the two age groups.

**Table 3:** Correlation between age group and the presentation of CAD and the angiographic severity of CAD CAD, coronary artery disease; ACS, acute coronary syndrome

Variable	Age: 20-40	Age: 41-60	P-value	Calculated χ²
	Value (%) (n = 89)	Value (%) (n = 180)		
Clinical presentation				
Stable CAD	40 (44.9%)	61 (33.9%)	0.08	3.06
ACS	49 (55.1%)	119 (66.1%)		
Angiographic severity (SYNTAX score)				
Mild CAD (SS of <23)	45 (50.6%)	79 (43.9%)	0.30	2.41
Moderate/severe CAD (SS of ≥23)	44 (49.4%)	101 (56.1%)		

Biochemical, echocardiographic, and angiographic findings

Biochemical and hematological parameters are summarized in Table 4. No significant differences were observed between the two groups in blood investigations, including lipid profile, complete blood count, liver function tests, and renal function tests. Echocardiographic assessment revealed similar mean left ventricular ejection fraction (LVEF) across groups. Angiographic analysis revealed that multivessel disease, complex lesion types (type C), and bifurcation lesions were more prevalent in the higher SYNTAX score group (all p < 0.001). Radial access was used in 97.0% of procedures overall, with femoral access slightly more common in the moderate/severe CAD group (p = 0.008). In the present study, mean free and total testosterone levels did not differ significantly between patients with ACS and those with stable CAD (Table 5). On age-stratified analysis, there was no statistically significant difference in either free or total testosterone levels between patients with mild CAD and those with moderate/severe CAD in both age groups (age of 20-40 years versus 41-60 years) (Table 6).

**Table 4:** Biochemical and hematological tests Hb, hemoglobin; TLC, total leucocyte count; DLC-N/L/M/E, differential leucocyte count: neutrophils/lymphocyte/monocyte/eosinophils; Hct, hematocrit; MCV, mean corpuscular volume; MCHC, mean corpuscular hemoglobin concentration; RDW, red cell distribution width; TG, triglyceride; Ct, count; SGOT, serum glutamic oxaloacetic transaminase; SGPT, serum glutamic pyruvic transaminase; SALP, serum alkaline phosphatase; RBS, random blood sugar; LDL-Chol, low-density lipoprotein cholesterol; HDL-Chol, high-density lipoprotein cholesterol; CAD, coronary artery disease; SD, standard deviation

Variable	Value (mean ± SD)	Mild CAD (mean ± SD)	Moderate to severe CAD (mean ± SD)	P-value	T-value
		(N = 124; 46.1%)	(N = 145; 53.9%)		
Hb (g/dL)	13.18 ± 1.88	13.22 ± 2.15	13.15 ± 1.61	0.76	0.19
TLC	8630 ± 2989	8480 ± 2733	8760 ± 3196	0.44	-0.63
DLC-N	60.39 ± 13.09	60.81 ± 13.50	60.03 ± 12.78	0.62	0.53
DLC-L	26.71 ± 8.47	26.10 ± 8.07	27.24 ± 8.79	0.27	-1.12
DLC-M	7.18 ± 2.60	7.27 ± 2.81	7.11 ± 2.41	0.61	0.47
DLC-E	3.95 ± 3.71	3.77 ± 3.61	4.10 ± 3.79	0.46	-0.55
Platelet Ct	186.39 ± 70.32	185.25 ± 63.95	187.37 ± 75.56	0.80	-0.22
Hct	41.66 ± 6.50	41.54 ± 7.07	41.77 ± 5.99	0.77	-0.20
MCV	90.00 ± 12.48	90.07 ± 12.06	89.94 ± 12.88	0.93	0.06
MCHC	31.85 ± 1.94	32.01 ± 1.58	31.72 ± 2.19	0.22	1.02
RDW	13.77 ± 4.07	14.01 ± 3.74	13.56 ± 4.34	0.36	0.74
Creatinine	1.06 ± 0.58	1.08 ± 0.67	1.03 ± 0.29	0.41	0.61
SGOT	48.04 ± 51.04	52.06 ± 56.98	44.61 ± 45.21	0.23	0.84
SGPT	44.92 ± 61.52	43.73 ± 29.25	45.94 ± 79.44	0.76	-0.28
SALP	95.70 ± 37.55	92.65 ± 29.86	98.30 ± 32.80	0.14	-1.23
RBS	145.25 ± 73.10	146.17 ± 77.67	144.47 ± 73.71	0.85	0.19
HbA1c	6.78 ± 1.76	7.06 ± 1.63	6.54 ± 1.96	0.42	1.63
Cholesterol (total)	136.55 ± 41.88	135.66 ± 43.32	137.32 ± 40.75	0.74	-0.29
LDL-Chol	82.57 ± 41.44	80.33 ± 31.41	84.49 ± 48.43	0.41	-0.79
HDL-Chol	35.17 ± 10.69	35.27 ± 12.87	35.09 ± 8.43	0.89	0.12
TG	148.20 ± 82.30	150.5 ± 93.89	146.26 ± 71.28	0.67	0.37

**Table 5:** Association between serum testosterone and the clinical presentation of CAD CAD, coronary artery disease; ACS, acute coronary syndrome; SD, standard deviation

Hormone levels	Diagnosis	N	Mean ± SD	T-value	P-value
Free testosterone	Stable CAD	101	27.5 ± 14	-1.26	0.22
	ACS	168	29.81 ± 15.34		
Total testosterone	Stable CAD	101	344.15 ± 166.81	0.93	0.35

**Table 6:** Association between serum testosterone and the angiographic severity of CAD in young and middle-aged male patients CAD, coronary artery disease; SD, standard deviation

Age group	Hormone levels	CAD severity	N	Mean ± SD	P-value	T-value
20-40	Free testosterone	Mild CAD	45	28.07 ± 14.41	0.53	0.64
		Moderate/severe CAD	44	26.27 ± 12.14		
	Total testosterone	Mild CAD	45	320.95 ± 156.15	0.84	0.20
		Moderate/severe CAD	44	314.4 ± 149.86		
41-60	Free testosterone	Mild CAD	79	28.66 ± 14.17	0.38	-0.90
		Moderate/severe CAD	101	30.72 ± 16.56		
	Total testosterone	Mild CAD	79	331.2 ± 163.82	0.58	-0.56
		Moderate/severe CAD	101	345.41 ± 174.87		

Six-month outcomes and MACE correlation with testosterone levels

Overall, MACE incidence was low at 3.3% (nine events in 269 patients). Specifically, there were three deaths during the follow-up period, five cases of non-fatal myocardial infarction for which repeat revascularization was done, and one case of cerebrovascular accident. The majority of repeat revascularization (four out of five) was done in patients with moderate/severe CAD. Patients who experienced MACE had lower mean free (22.94 ± 7.47 pg/mL) and total testosterone levels (257.98 ± 157.42 pg/mL) compared to those without events (29.19 ± 15.05 pg/mL and 335.23 ± 164.05 ng/dL, respectively), though the differences were not statistically significant (Table 7).

**Table 7:** MACE at six months Major adverse cardiovascular events (MACE): a composite of cardiovascular death, non-fatal MI, target vessel revascularization, and stroke ¶The Mann-Whitney U test MI: myocardial infarction

Testosterone levels	MACE		P-value	T-value
	MACE present (n = 9)	MACE absent (n = 260)		
Free testosterone	22.94 ± 7.47	29.19 ± 15.05	0.17	-2.35¶
Total testosterone	257.98 ± 157.42	335.23 ± 164.05	0.13	-1.45

Total testosterone levels were lower in individuals with diabetes compared to those without, suggesting a possible inverse link. However, free testosterone did not show this trend. Other risk factors, such as hypertension, dyslipidemia, smoking, alcohol use, sedentary lifestyle, family history of CAD, and diet preference (vegetarian or non-vegetarian), showed no noticeable correlation with either free or total testosterone levels (Table 8).

**Table 8:** Correlation of serum testosterone with CAD risk factors HTN, hypertension; DM, diabetes mellitus; h/o, history of; CAD, coronary artery disease

Variable	Testosterone (free)	P-value (free)	T-value	Testosterone (total)	P-value (total)	T-value
Smoking		0.84	-0.20		0.38	-1.02
No (n = 164)	28.80 ±14.18			325.11 ± 162.38		
Yes (n = 105)	29.18 ±15.95			342.94 ± 167.24		
Alcohol		0.82	-0.23		0.48	-0.73
No (n = 203)	28.89 ± 14.99			328.34 ± 153.49		
Yes (n = 66)	29.37 ± 15.19			344.58 ± 189.59		
HTN		1.00	0.00		0.11	1.32
No (n = 204)	28.94 ± 15.13			341.07 ± 156.57		
Yes (n = 65)	28.94 ± 14.10			303.84 ± 184.66		
DM		0.51	0.65		0.01	2.62
No (n = 188)	29.33 ± 14.56			347.63 ± 158.66		
Yes (n = 81)	28.03 ± 15.63			295.31 ± 172.13		
Dyslipidemia		0.44	0.78		0.40	0.76
No (n = 245)	29.16 ± 14.91			334.70 ± 161.71		
Yes (n = 24)	26.73 ± 14.49			305.22 ± 189.61		
Family h/o CAD		0.16	1.42		0.38	-0.80
No (n = 195)	29.72 ± 15.63			326.64 ± 162.63		
Yes (n = 74)	26.89 ± 12.50			346.37 ± 168.60		
Sedentary lifestyle		0.52	1.16		0.84	-0.48
No (n = 175)	29.37 ± 15.43			330.62 ± 164.94		
Yes (n = 94)	28.14 ± 13.79			334.77 ± 163.70		
Non-vegetarian diet		0.25	1.18		0.15	-1.50
No (n = 107)	30.21 ± 15.21			314.32 ± 153.52		
Yes (n = 162)	28.10 ± 14.61			343.79 ± 170.36		

## Discussion

This prospective observational study evaluated the association between serum testosterone levels and the angiographic severity of CAD in young and middle-aged Indian men. Despite prior evidence suggesting a link between low testosterone levels and greater atherosclerotic burden, the present study found no significant difference in either total or free testosterone levels between patients with mild (SYNTAX of <23) and those with moderate/severe disease (SYNTAX of ≥23). Furthermore, there was no meaningful correlation between testosterone levels and SYNTAX score, with correlation coefficients for total and free testosterone being -0.0008 and -0.0179, respectively. These findings suggest that serum testosterone does not independently predict angiographic severity in this population.

Our results contrast with some earlier studies that proposed an inverse relationship between testosterone and coronary artery disease severity. Phillips et al. first reported a significant negative correlation between testosterone levels and angiographically proven CAD, independent of age and adiposity [3]. More recently, studies done by Li et al. [6] and Hu et al. [7] demonstrated an inverse relationship between serum testosterone levels and Gensini scores. A South Indian study by Gopalakrishnan et al. found that lower testosterone levels were linked to increased CAD risk, suggesting a protective vascular role [5]. Similarly, Gururani et al. reported lower testosterone levels in patients with CAD, with an inverse correlation between testosterone and disease severity assessed by the Gensini score [4].

Certain earlier studies have not demonstrated a consistent association between serum testosterone levels and CAD [8-10]. In contrast, some studies have proposed a nonlinear association between testosterone levels and CAD, suggesting that while lower levels may confer a protective effect, excessively high levels could paradoxically increase cardiovascular risk [11]. No study to date has found an association between higher serum testosterone concentrations and CAD. However, Soisson et al. have shown that a J-shaped curve exists for a combined end point of ischemic stroke and myocardial infarction (MI), with the first and fifth quintiles of serum testosterone having comparatively greater event rates than the second quintile [12]. The conflicting evidence of testosterone levels and CAD likely reflects the complex pathophysiology of the disease.

Our study contributes to this complex and evolving narrative by providing data from a North Indian population and showing no significant link between testosterone levels and angiographic disease severity. The absence of a significant relationship in our study may be explained by several factors. First, our cohort comprised relatively young men, and testosterone levels in this group may not reflect the hormonal derangements seen in older populations. Second, many patients were already on cardiovascular medications, including statins and beta-blockers, which may influence testosterone metabolism and mask its vascular effects. Additionally, our study also included patients with ACS, in whom the hormonal milieu may be transiently altered due to acute stress and systemic inflammation, potentially acting as a confounding factor in assessing baseline testosterone levels. Acute systemic inflammation and elevated cytokine levels during ACS are known to suppress the hypothalamic-pituitary-gonadal axis and reduce circulating testosterone concentrations [13,14]. This transient hypogonadal state may obscure baseline hormonal relationships and contribute to the absence of a clear association between testosterone levels and CAD severity observed in our study.

We compared patients in two age groups, 20-40 years and 41-60 years. ACS was more common in the older group, but this difference was not statistically significant. The angiographic severity of CAD was also similar between the two age groups. Testosterone levels did not show any significant variation with the severity of CAD, even when younger and older patients were analyzed separately. This suggests that age does not influence the relationship between testosterone and CAD severity. Likewise, testosterone levels were not significantly different between patients presenting with stable CAD and those with ACS, indicating that whether the presentation was acute or chronic did not affect testosterone levels in our study.

In terms of clinical outcomes, testosterone levels were comparable between patients who developed major adverse cardiovascular events (MACE) during follow-up and those who did not. This suggests that testosterone levels may not serve as reliable short-term prognostic biomarkers in patients with CAD. It is important to note that MACE incidence was too low (3.3%) in the present cohort to make any meaningful prognostic analysis of the impact of testosterone levels on short-term outcomes.

In our study, diabetic patients had lower serum total testosterone levels compared to non-diabetics, pointing toward a possible inverse relationship between testosterone and diabetes. Previous studies have shown that older diabetic men tend to have lower endogenous androgen levels, which are also linked to diabetic dyslipidemia [15]. For other conventional CAD risk factors, including hypertension, smoking, dyslipidemia, alcohol use, sedentary lifestyle, and family history of premature CAD, testosterone levels remained broadly similar across groups. These findings suggest that while diabetes may be associated with lower testosterone levels, the hormone does not appear to vary significantly with other traditional cardiovascular risk factors in our cohort. This reinforces the idea that the relationship between testosterone and CAD is likely multifactorial and may not be strongly influenced by individual risk factors alone.

Our study has several limitations. It was a single-center study with a modest sample size, limiting generalizability. Hormone levels were measured only once, and diurnal or longitudinal variations were not accounted for. Furthermore, we did not include a control group without CAD, which could have provided estimates of testosterone levels in healthy men of a similar age profile. Also, we were unable to recruit the planned number of patients in the stable CAD group, specifically younger patients aged between 20 and 40 years. As a result, the total sample size was reduced to 269 instead of the intended 296. This shortfall, particularly in the stable CAD subgroup, may have affected the statistical power of the study, limiting our ability to detect significant differences or associations, especially in subgroup analysis. The majority of patients had presented with ACS, and acute-phase hormonal changes in testosterone levels in patients with ACS could have affected the results of the study. Low MACE rate (3.3%) limits the power for the prognostic analysis of this study. Finally, since the study was conducted in a tertiary care center, patients with more severe disease may have been overrepresented, introducing referral bias.

Despite these limitations, the study has notable strengths. It is among the few prospective studies examining the testosterone-CAD relationship in an Indian population, with standardized hormonal assessments and angiographic scoring. The inclusion of both stable patients and patients with ACS, as well as patients in the age groups 20-40 and 41-60 years, was done to include the whole spectrum of young and middle-aged men with CAD. Moreover, our negative findings are still valuable, as they challenge assumptions drawn from previously published literature and reinforce the importance of population-specific research.

In interpreting these findings, it is important to consider the possibility that testosterone-CAD relationships are context-dependent and may be influenced by population-specific factors. In South Asian men, the high prevalence of conventional risk factors such as diabetes and dyslipidemia may overshadow the potential vascular effects of endogenous testosterone, reducing its apparent predictive value. Moreover, the inclusion of patients with acute coronary syndrome could have attenuated associations due to transient hormonal changes during acute illness. The lack of a significant difference across SYNTAX score strata suggests that, at least in this cohort, structural coronary disease burden may be more strongly determined by traditional metabolic and inflammatory pathways than by androgen status. Future multicenter studies with larger sample sizes, repeated hormone measurements, and the inclusion of a healthy control arm would help clarify whether testosterone measurement adds incremental value to CAD risk stratification in the Indian male population.

## Conclusions

In this prospective study of young and middle-aged Indian men with angiographically confirmed coronary artery disease, serum total and free testosterone levels were not significantly associated with disease severity or short-term cardiovascular outcomes. Notably, although diabetic patients exhibited lower total testosterone levels, this did not translate into measurable differences in CAD severity or outcomes, highlighting the multifactorial nature of cardiovascular risk. This study is unique in integrating detailed hormonal assessment with objective angiographic scoring in an Indian cohort, a population where data on endogenous testosterone and coronary atherosclerosis are scarce despite a high burden of premature CAD. Our findings suggest that single-point testosterone estimation may have limited value for cardiovascular risk stratification, highlighting the need for larger, longitudinal studies with serial hormone measurements to define its prognostic relevance.
